# Efficacy of combined transbronchial lung cryobiopsy and conventional forceps biopsy for lung malignancies: a prospective cohort study

**DOI:** 10.1038/s41598-023-29007-y

**Published:** 2023-02-01

**Authors:** Kohei Kinoshita, Kei Morikawa, Hajime Tsuruoka, Motohiro Chosokabe, Hirotaka Kida, Hiroshi Handa, Takeo Inoue, Tomoyuki Miyazawa, Hisashi Saji, Masamichi Mineshita

**Affiliations:** 1 Division of Respiratory Diseases, Department of Internal Medicine, St. Marianna University School of Medicine, 2-16-1 Sugao, Miyamae-ku, Kawasaki, 216-8511 Japan; 2 Department of Chest Surgery, St. Marianna University School of Medicine, Kawasaki, Kanagawa Japan; 3 Department of Pathology, St. Marianna University School of Medicine, Kawasaki, Kanagawa Japan

**Keywords:** Lung cancer, Lung cancer

## Abstract

There are few prospective reports of transbronchial lung cryobiopsy (TBLC) for malignant tumors in combination with forceps biopsy. We investigated the clinical parameters in which TBLC is superior to forceps biopsy. This is a prospective cohort study to analyse the efficacy of TBLC for suspected malignancy. TBLC was performed after brushing cytology and forceps biopsy, and the diagnostic yield for TBLC, brushing cytology, and forceps biopsy were examined. Adverse events were defined as those requiring additional procedures. Next-generation sequencing (NGS) analysis was performed in each case of non-small cell lung cancer. Of the 100 patients, malignancy was confirmed in 94 cases. The diagnostic yield for TBLC/forceps biopsy/brushing cytology was 86/81/82% respectively, while the diagnostic yield for all procedures combined was 94%. There was no significant difference in the diagnostic yield between TBLC and forceps biopsy. When comparing the biopsy site, the diagnostic yield for TBLC at the lower lobe was significantly higher than forceps biopsy (P < 0.01). Endobronchial ultrasonography imaging using a guide-sheath did not significantly differ in the diagnostic yield of TBLC. The success rate of NGS for TBLC specimens was 100% (26 cases). Adverse events included two cases of severe bleeding. TBLC of peripheral lesions may improve the diagnostic yield when combined with forceps biopsy and brushing cytology. The diagnostic yield of TBLC was higher at the lower lobes. Furthermore, TBLC provided sufficient specimen quality for NGS.

## Introduction

Personalized medicine for lung cancer using molecular-targeted drugs and immune checkpoint inhibitors have become widespread. Still, it is necessary to collect sufficient specimens to determine treatment strategies for each case^1,2^. Furthermore, new genetic mutations and their corresponding molecular target drugs are expected in the future. Individualized treatment based on programmed death-ligand 1 (PD-L1) expression rate and pathological subtypes are also expected, thereby increasing specimens required for each test^3^.

Generally, conventional bronchoscopic specimens are small, and it is difficult to prove malignant findings including genetic diagnosis in some cases. With the widespread use of transbronchial lung cryobiopsy (TBLC), the collection of specimens which are several times larger in area are possible^4,5^. TBLC has been used mostly for interstitial pneumonia, but its usefulness in malignant disease is not yet fully known.

Pathologically, specimens of forceps biopsy are significantly smaller than those collected by TBLC. In addition to the smaller volume of specimens, high quality specimens cannot always be obtained due to crush artifacts by forceps biopsy. In a previous report, the evaluable site and the amount of DNA and RNA of each specimen was significantly larger for frozen specimens, which may be superior to forceps biopsy in the diagnosis of lung cancer histology^6,7^. However, the diagnostic factors favouring TBLC over forceps biopsy remain unclear except for specimen size. Previous studies have reported that TBLC is a beneficial procedure in cases with adjacent to findings on endobronchial ultrasonography (EBUS)^8^, and in peripheral lesions with a longest diameter of 22 mm or less on CT findings^9^, but no consensus has been reached on which cases should be preferred for TBLC.

Additionally, non-small cell lung cancer (NSCLC) may have driver mutations, including *EGFR*, *ALK*, *ROS1*, *RET* and *BRAF*^10,11^ . Furthermore, next-generation sequencing (NGS), such as the Oncomine™ Dx Target Test^®^ (ODxTT), to measure multiple gene mutations simultaneously have been used as a companion diagnostic test, but the test failure rate of ODxTT was reported to be up to 28%^12^. However, two factors can increase the success rate of ODxTT: (1) volume of specimens and (2) high tumor nuclei content. Therefore, TBLC might be useful for NGS^13^. For these reasons, TBLC is expected to become more widespread in the future. Furthermore, only few studies have prospectively examined the side effects of TBLC. We compared the diagnostic accuracy between TBLC and forceps biopsy, and examined the incidence of adverse events.

## Methods

This prospective, cohort study was performed in single center from January 2020 to September 2021. A total of 217 patients were referred to our hospital to perform bronchoscopy with peripheral lung lesion which were suspected of lung cancer. Of these patients, with no contraindications, underwent a TBLC. The contraindications for TBLC were defined as follows: severe hypoxemia (PaO_2_ (partial pressure of oxygen) < 60 mmHg) or hypercapnia (PaCO_2_ (partial pressure of carbon dioxide ) > 50 mmHg), bleeding disorders, large vessels near the tumor on the CT, technically difficult to introduce cryoprobe, excessive bleeding after forceps biopsy, which was needed for extra intervention to stop bleeding.

The trial was approved by the medical ethics committee, clinical research section at St. Marianna University (Case No: 4755) and was registered within UMIN Clinical Trials Registry (UMIN000039618, first posted 27/02/2020). All methods were performed in accordance with the declaration of Helsinki.

The bronchoscopes used in this study were: (1) BF-1T260, a standard bronchoscope with high procedural versatility and image quality, (2) BF-P260F, which is thinner and able to reach peripheral sites, and (3) BF-6C260, a bronchoscope with high image quality. (Olympus Marketing Corp., Tokyo, Japan).

In all patients, tracheal intubation (SASSET tracheal tube 8 mm, ICU Medical, CA, USA) was performed using a bronchoscope with a connector (AQUA + TS, Teleflex Medical Sdn Bhd, Perak, Malaysia), to administer oxygen. Radial EBUS with guide-sheath (K-201, or K-203 guide-sheath kit, Olympus Marketing Corp., Tokyo, Japan) was used for all cases except when the cryoprobe could not be inserted through a guide sheath or when the bronchoscope could reach the lesion directly.

Bronchoscopy was performed under laryngeal anaesthesia with 4% xylocaine, followed by sedation with midazolam and analgesia with fentanyl. After confirming the target lesion using EBUS image and fluoroscopy, we underwent an average of 3 passes for brushing cytology and forceps biopsy in any order, followed by TBLC using a cryoprobe with a diameter of 1.9 mm in most situations (Erbe CRYO2, Erbe Elektromdizin GmbH, Tübingen, Germany). TBLC was usually performed once, and additional biopsies were performed if the specimen volume was insufficient. Prior to the TBLC, a Fogarty balloon catheter (Forgarty catheter, Edwards Lifesciences Corp., Ca, USA) was placed in the access bronchus, and balloon dilation was performed immediately after pulling out the cryoprobe.

After examinations, chest X-ray was taken to confirm complications, such as pneumothorax or bleeding. Severe adverse events were defined as requiring additional treatments or procedures (e.g., tracheal intubation, ventilation, surgical treatment, or embolization)^6^.

### Pathological diagnosis

The collected specimens were cytopathologically analysed by a cytologist and at least two pathologists. The area of the histological specimen by TBLC was measured at maximum split plane by cellSens Standard software (cellSens Standard v.1.7, EVIENT, Tokyo, Japan). Tumor nuclei content was determined graphically by a pathologist, and was evaluated three times, three days apart, with outliers excluded.

In the case of NSCLC, NGS analysis was performed using ODxTT. The success of ODxTT was defined as a positive or negative result for four genetic mutation tests: *EGFR, ALK, ROS1* fusion gene, and *BRAF,* which were performed as a companion diagnosis at the time of this study. Unsuccessful results were defined as invalid or no call. Molecular testing of the single-plex test was performed in a part of cases diagnosed as lung adenocarcinoma by pathological evaluation. The Roche cobas^®^ EGFR mutation test v2 (Roche Molecular Systems, Pleasanton, CA, USA), was used for testing EGFR mutations. Unsuccessful results were defined as invalid or failed. Vysis ALK Break Apart FISH Probe Kit (Abbott Molecular, Des Plaines, IL, USA) was used for testing ALK mutations.

Cytological evaluation was based on Papanicolaou's classification, with Class III or higher judged to be diagnosable. Pathologists evaluated the number of tumor cells and tumor nuclei content. The tumor nuclei content was calculated as the percentage of tumor cell nuclei among the nucleated cells in the tissue section.

Statistical analysis was performed using EZR on R commander ver. 1.56. A comparison of forceps biopsy and TBLC for each condition was performed using the McNemar test or the Pearson’s Chi-squared test.

### Ethics approval and consent to participate

The clinical protocol was approved by the institutional review board. All patients provided written informed consent before study entry.

## Results

### Patients and diagnosis

**Figure 1:**
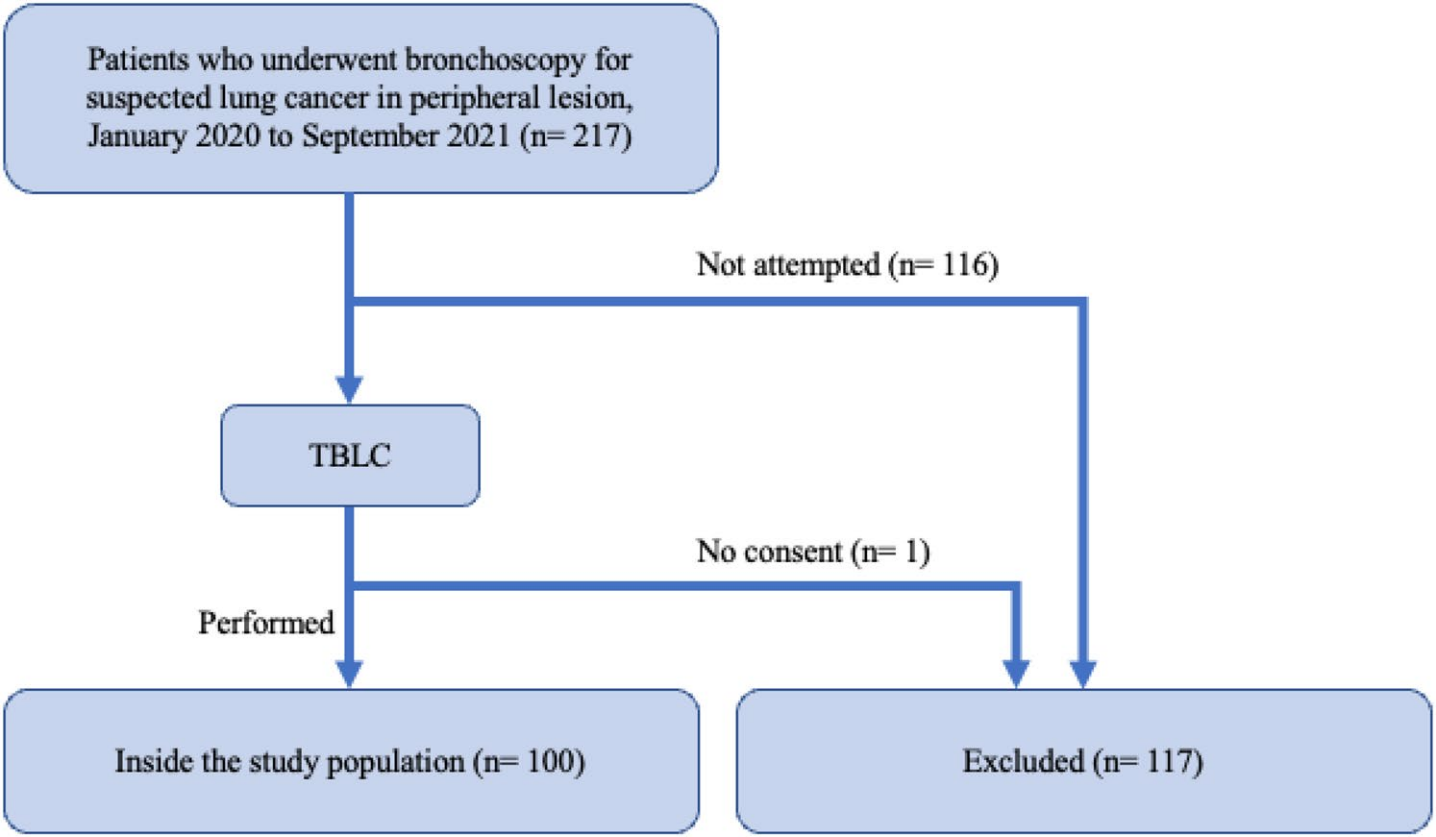
Flow diagram displaying patient selection.

TBLC were performed in 101 patients. One person could not give consent and was excluded. A total of 100 patients participated in our study (Fig. 1). The median age was 74 years (range; 41–91), and 62 (62%) were male (Table 1). Table 2 shows the diagnostic results of all patients. Bronchoscopy confirmed malignancies in 94 cases (94%), with adenocarcinoma accounting for 59 cases (59%). Forceps biopsy was not performed in two cases due to time restraints.

**Table 1:** Patient and lesion characteristics.

Patient characteristics, *n* (%)	
Male/female	62 (62%)/38 (38%)
Median age, (range)	74 yr, (41–91)
Smoking history: current and ex-smoker	72 (72%)
Lesion characteristics, *n* (%)	
Median tumor diameter (range)	37 mm (11–127)
Lobar location	
Upper lobe	55 (55%)
Middle lobe	8 (8%)
Lower lobe	37 (37%)
EBUS-GS	
With GS	80 (80%)
Without GS	20 (20%)
EBUS B-mode image	
Within	87 (87%)
Within—blizzard	2 (2%)
Adjacent to	11 (11%)

*EBUS* endobronchial ultrasonography, *GS* guide sheath.

**Table 2:** Morphological diagnosis from TBLC.

Pathological diagnosis from TBLC, *n* (%)	
Adenocarcinoma	59 (59%)
Squamous cell carcinoma	19 (19%)
SCLC	3 (3%)
NSCLC, NOS	2 (2%)
Carcinoma	4 (4%)
Metastatic cancer	2 (2%)
LCNEC	1 (1%)
Malignant lymphoma	1 (1%)
Plasma cell tumor	1 (1%)
AAH	1 (1%)
Non-diagnostic	6 (6%)

*TBLC* transbronchial lung cryobiopsy, *SCLC* small cell lung cancer, *NSCLC* non-small cell lung cancer, *NOS* not otherwise specified, *LCNEC* large-cell neuroendocrine carcinoma, *AAH* atypical adenomatous hyperplasia.

### Diagnostic yield

The mean tumor diameter using computed tomography (CT) images was 37 mm (range 11–127 mm), and the average number of biopsies was 2.4 for forceps biopsy and 1.1 for TBLC, activated for 5.0 s (range; 3–7 s) of mean freezing time. The mean sample size of TBLC was 9.77 mm^2^ (range 0.86–44.31 mm^2^) (Table 3).

**Table 3:** Pathological sample characteristics from TBLC.

Pathological sample characteristics from TBLC	
Median size (range, 95% CI)	9.77 cm^2^ (9.80–12.97)
Median tumor nuclei content (range)	50% (10–90)
Number of tumor cells	
< 300	17 (19.8%)
300–500	18 (20.9%)
500 <	51 (59.3%)

TBLC diagnosed 86 cases (86%), forceps biopsy diagnosed 81 of 98 cases (82.7%), and cytology diagnosed 82 cases (82%) (Table 4). The number of cases diagnosed by a single procedure alone was 1, 2, and 5 cases for TBLC, forceps, and brushing, respectively. The diagnostic yield increased to 94% when all procedures were combined. Six cases were unable to be diagnosed by all techniques (6.0%). There was no significant difference in the diagnostic yield between TBLC and forceps biopsy (P = 0.206).

**Table 4:** Diagnostic yield for each condition.

Combined diagnostic yield				
94/100 (94%)				
Diagnostic rate by technique				
	Brushing cytology	Forceps biopsy	Cryobiopsy	P value
	82/100 (82.0%)	81/98 (82.7%)	86/100 (86.0%)	P = 0.34
Diagnostic yield by location				
RUL and LUS	44/55 (80.0%)	45/53 (84.9%)	44/55 (80.0%)	P = 0.48
RML and lingula	7/8 (87.5%)	8/8 (100%)	7/8 (87.5%)	N/E
RLL and LLL	31/37 (83.3%)	28/37 (75.7%)	35/37 (94.5%)	P = 0.023
Diagnostic yield by EBUS B-mode image				
Within	73/87 (83.9%)	73/87 (83.9%)	76/87 (87.4%)	P = 0.505
Adjacent to	9/11 (81.8%)	7/10 (70.0%)	9/11 (81.8%)	P = 1.0
Within-blizzard	0/2 (0%)	1/1 (100%)	1/2 (50.0%)	N/E
Diagnostic yield by guide sheath				
With GS				
Total	66/80 (82.5%)	66/78 (84.6%)	71/80 (88.8%)	P = 0.29
Upper lobe	33/42 (78.6%)	35/40 (87.5%)	35/42 (83.3%)	P = 1.0
Middle lobe	6/7 (85.7%)	7/7 (100%)	6/7 (85.7%)	N/E
Lower lobe	27/31 (87.1%)	24/31 (77.4%)	30/31 (96.8%)	P = 0.041
Without GS	–	–	15/20 (75.0%)	

Data are presented as *n* (%).

Two cases did not undergo forceps biopsy at the upper lobe.

*N/E* not evaluated, *EBUS* endobronchial ultrasonography, *GS* guide sheath, *LLL* left lower lobe, *LUS* left upper segment, *RLL* right lower lobe, *RML* right middle lobe, *RUL* right upper lobe.

The location of biopsies and the diagnostic yield for each procedure are shown in Table 4. In the upper lobe, the diagnostic yield for brushing cytology and TBLC was 80.0% (44/55, each), while forceps biopsy was 84.9% (45/53). There was no significant difference in the diagnostic yield between forceps biopsy and TBLC. In the middle/lingular lobe, the diagnostic yield for brushing cytology and TBLC was 87.5% (7/8 each), while forceps biopsy was 100% (8/8). In the lower lobe, the diagnostic yield of brushing cytology was 83.8% (31/37), forceps biopsy was 75.7% (28/37), and TBLC was 94.5% (35/37). The diagnostic yield for TBLC was significantly higher than that of forceps biopsy in the lower lobe (P = 0.0082).

Table 4 shows echogenic findings by radial EBUS and the diagnostic yield of each technique. When echo findings were within the lesion, the diagnostic yield for brushing cytology and forceps biopsy were both 83.9% (73/87), while TBLC was 87.4% (76/87). When echo findings were adjacent to the lesion, the diagnostic yield for brushing cytology was 81.8% (9/11), while forceps biopsy was 70.0% (7/10). The diagnostic yield for TBLC was 81.8% (9/11). When echo findings visualized mixed blizzard signs in two cases, the diagnostic yield of brushing cytology was 0% (0/2), forceps biopsy was 100% (1/1), and TBLC was 50.0% (1/2).

Table 4 also shows the diagnostic yield for each procedure and location in cases where GS was used. The total diagnostic yield for brushing cytology with GS was 82.5% (66/80). For the brushing cytology subgroup, the diagnostic yield for the upper lobe, middle/lingular lobe, and lower lobe was 78.6% (33/42), 85.7% (6/7), and 87.1% (27/31), respectively. The total diagnostic yield for forceps biopsy with GS was 84.6% (66/78). For the forceps biopsy subgroup, the diagnostic yield of the upper lobe, middle/lingular lobe, and lower lobe was 87.5% (35/40), 100% (7/7), and 77.4% (24/31), respectively. The total diagnostic yield for TBLC with GS was 88.8% (71/80). For the TBLC subgroup, the diagnostic yield of the upper lobe, middle/lingular lobe, and lower lobe was 83.3% (35/42), 85.7% (6/7), and 96.8% (30/31), respectively.

The diagnostic yield of TBLC without GS was 75% (15/20). There was no significant difference in the diagnostic yield for TBLC with or without GS (P = 0.145).

### Adverse events

There were two cases of severe bleeding during TBLC. In one case, a Fogarty balloon catheter was effective but active bleeding continued after deflation of the balloon. An endobronchial Watanabe spigot was placed to stop the persistent bleeding. In the second case, active bleeding with a volume of about 500 ml was suctioned immediately after TBLC due to the balloon catheter displacement. This patient suffered from hypoxemia and hypotension. After haemostasis by wedging the bronchoscope to the access bronchus and changing to the side lying position, the patient recovered from hypoxia and was transferred to the intensive care unit and extubated the following day.

### Next-generation sequencing (NGS) and single-plex molecular test

The success rate of NGS for TBLC samples was 100% (26/26). Of the 26 patients who underwent NGS, 18 cases had a tumor cell count of 500 or more, and 8 cases had a cell count of less than 500. The median tumor nuclei content in all TBLC cases was 50% (10–90%). Tumor nuclei content was less than 30% and 20% in 6 (6%) and 3 (3%) cases, respectively. The single-plex test was used in 32 cases of cobas^®^ and 3 cases of FISH, all of which were successfully performed.

## Discussion

We revealed that TBLC for peripheral lesions had a diagnostic yield as high as both forceps biopsy and brushing cytology. It should be noted that when all techniques were combined, a diagnostic yield of 94.0% was observed. Whereas the diagnostic yield of each technique alone was around 85% in our study, with no significant difference observed between techniques. A previous study reported that combining all techniques resulted in a high diagnostic yield of 89.9%, but the diagnostic yield of TBLC alone was rather inferior to that of conventional biopsy (74.3% vs. 81.3%)^8^. Therefore, each technique has its advantages and disadvantages, and it is presumed that combining the procedures complements the diagnostic yield. For this reason, TBLC should be performed in combination with conventional biopsy.

We investigated the characteristics of each lesion, and the efficacy of TBLC was compared with forceps biopsy. Although previous reports have shown that the diagnostic yield of TBLC was higher in patients with EBUS findings adjacent to lesions than forceps biopsy^7,8^, no significant difference was observed in our study. This might be because there were only 11 cases where EBUS findings were adjacent to the lesion. Although the cryoprobe has the ability to freeze specimens circumferentially, further case accumulation is required to evaluate the usefulness of the cryoprobe for EBUS-adjacent lesions.

In subgroup analysis by lesion site, the diagnostic yield for TBLC was significantly higher than that of forceps biopsy at the lower lobes. However, previous reports stated no difference in diagnostic yield depending on the biopsy location^7,14^. Empirically, lower lobe lesions are subject to fluctuations in biopsy location due to respiratory variability. However, as the cryoprobe instantly adheres to the lesion by freezing, the displacement risk of the lesion site due to respiratory variability may be reduced.

**Figure 2:**
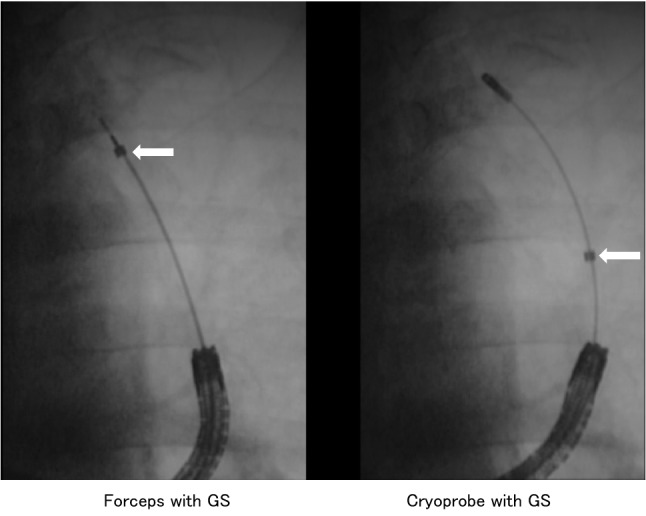
Guide sheath displaced due to cryoprobe insertion. Figure 1 shows one case of GS displacement by the TBLC probe insertion for a lesion at the lung apex. White arrows indicate GS tips. The tip of the GS is displaced by the insertion of the cryoprobe and is significantly different from the site where the forceps biopsy was performed. *GS* guide sheath.

Generally, TBLC at the upper lobe is considered to be difficult to perform, and various techniques have been reported^8,15^. In our study, the diagnostic yield of TBLC at the upper lobe was 80.0%, and there was no significant difference between the upper and other lobes. Primary lung cancer is known to frequently affect the upper lobe with previous studies showing a diagnostic yield of 77.8%, which was similar to our study^16^. However, we found one case of GS displacement by the TBLC probe insertion for a lesion at the lung apex (Fig. 2). In this case, forceps biopsy proved malignant cells, while TBLC showed a negative result, which may indicate a limitation for TBLC with GS. However, as a technique for guiding the cryoprobe more smoothly and accurately, an injection of olive oil into the GS can more easily guide the cryoprobe to the lung periphery^16^. In the middle/lingular lobe, the diagnostic yield was high for all procedures but the number of cases was low, and further study is needed.

In our study, the diagnostic yield of TBLC with and without GS were 88.8% (71/80) and 75.0% (15/20), respectively. It has been reported that TBLC with GS had a significantly higher diagnostic yield than TBLC without GS^17^. However, a simple comparison was difficult due to the imbalance of cases in our study.

We described two cases of severe bleeding. The incidence rate of adverse events in our study was similar to previous reports^18^. A previous study reported that radial EBUS imaging can reduce the risk of bleeding by searching for blood vessels at the biopsy site^19^. Although we performed the same procedure using the EBUS-GS technique, severe bleeding could not be prevented in all cases. Routine preoperative screening based on medical history and blood sample results are not sensitive enough to detect all factors related to bleeding; especially in rare diseases with primary haemostatic abnormalities such as von Willebrand's disease^20^. Our two patients with severe bleeding also had no abnormalities on preoperative tests. A previous study reported that histological factors, such as renal cell carcinoma, can predict severe bleeding^21^. Furthermore, CT findings suggested that lesions in the proximal 1/3 of the hilar region are at risk for severe bleeding^22^. None of these factors were present for our 2 cases.

The NGS success rate was 100%. Generally, the tumor nuclei content required for NGS is reported to be at least 30%^23,24^. The median tumor nuclei content in our study was 50%, which was sufficient for NGS. Additionally, tumor cell necrosis may have been a factor for unsuccessful NGS results, but EBUS-GS can be used to avoid biopsies in areas with necrotic lesions^13,25^. The combination of EBUS in our study may have resulted in the high NGS success rate.

There were some limitations to our study. First, this was a single-centre study and therefore, further study with larger populations are needed to confirm these results. Second, performing TBLC as the final procedure may cause bleeding due to forceps biopsy or other previous procedures. The possibility cannot be ruled out that bleeding from brush or forceps biopsy may have affected the diagnostic rate of TBLC in this study. Furthermore, the number of passes for each procedure and the TBLC freezing time were not equal, which may have affected the diagnostic yield. Finally, it is not clear if our results could apply to thinner, disposable cryoprobes that could be commonly used in the future^16^. In particular, the disposable cryoprobe's flexibility allows the use of a narrow bronchoscope to approach the apex of the lung, which has a strong curvature, and its combined use with conventional biopsy has been reported to increase the diagnostic yield^9,26,27^. In addition, it may be useful to combine the peripheral cryoprobe approach with new technologies such as F-ENB and robotic assist, and this should be investigated as well^28,29^.

## Conclusion

TBLC for a peripheral lung lesion is usually combined with forceps biopsy and brushing cytology to improve the diagnostic yield. The diagnostic yield for TBLC in this study was higher than that of forceps biopsy, especially for lower lobe lesions. Furthermore, TBLC was able to provide sufficient quality and quantity of specimens for NGS panel tests.

## Data Availability

The datasets used and/or analyzed during the current study are available from the corresponding author on reasonable request.

## Abbreviations

CT: Computed tomography
EBUS: Endobronchial ultrasonography
GS: Guide sheath
NGS: Next generation sequencing
PD-L1: Programmed death-ligand 1
TBLC: Transbronchial lung cryobiopsy

## References

[1] Maemondo M, Inoue A, Kobayashi K, Sugawara S, Oizumi S, Isobe H, Gemma A, Harada M, Yoshizawa H, Kinoshita I (2010). Gefitinib or chemotherapy for non-small-cell lung cancer with mutated EGFR. N. Engl. J. Med..

[2] Solomon BJ, Mok T, Kim D-W, Wu Y-L, Nakagawa K, Mekhail T, Felip E, Cappuzzo F, Paolini J, Usari T (2014). First-line crizotinib versus chemotherapy in *ALK*-positive lung cancer. N. Engl. J. Med..

[3] Jordan EJ, Kim HR, Arcila ME, Barron D, Chakravarty D, Gao J, Chang MT, Ni A, Kundra R, Jonsson P (2017). Prospective comprehensive molecular characterization of lung adenocarcinomas for efficient patient matching to approved and emerging therapies. Cancer Discov..

[4] Babiak A, Hetzel J, Krishna G, Fritz P, Moeller P, Balli T, Hetzel M (2009). Transbronchial cryobiopsy: A new tool for lung biopsies. Respiration.

[5] Schumann C, Hetzel J, Babiak AJ, Merk T, Wibmer T, Möller P, Lepper PM, Hetzel M (2010). Cryoprobe biopsy increases the diagnostic yield in endobronchial tumor lesions. J. Thorac. Cardiovasc. Surg..

[6] Hetzel J, Eberhardt R, Herth FJF, Petermann C, Reichle G, Freitag L, Dobbertin I, Franke KJ, Stanzel F, Beyer T (2012). Cryobiopsy increases the diagnostic yield of endobronchial biopsy: A multicentre trial. Eur. Respir. J..

[7] Udagawa H, Kirita K, Naito T, Nomura S, Ishibashi M, Matsuzawa R, Hisakane K, Usui Y, Matsumoto S, Yoh K (2020). Feasibility and utility of transbronchial cryobiopsy in precision medicine for lung cancer: Prospective single-arm study. Cancer Sci..

[8] Matsumoto Y, Nakai T, Tanaka M, Imabayashi T, Tsuchida T, Ohe Y (2021). Diagnostic outcomes and safety of cryobiopsy added to conventional sampling methods: An observational study. Chest.

[9] Kim SH, Mok J, Jo EJ, Kim MH, Lee K, Kim KU, Park HK, Lee MK, Eom JS (2022). The additive impact of transbronchial cryobiopsy using a 1.1-mm diameter cryoprobe on conventional biopsy for peripheral lung nodules. Cancer Res. Treat..

[10] Kohno T, Ichikawa H, Totoki Y, Yasuda K, Hiramoto M, Nammo T, Sakamoto H, Tsuta K, Furuta K, Shimada Y (2012). KIF5B-RET fusions in lung adenocarcinoma. Nat. Med..

[11] Saito M, Shiraishi K, Kunitoh H, Takenoshita S, Yokota J, Kohno T (2016). Gene aberrations for precision medicine against lung adenocarcinoma. Cancer Sci..

[12] Planchard D, Besse B, Groen HJM, Souquet P-J, Quoix E, Baik CS, Barlesi F, Kim TM, Mazieres J, Novello S (2016). Dabrafenib plus trametinib in patients with previously treated BRAFV600E-mutant metastatic non-small cell lung cancer: an open-label, multicentre phase 2 trial. Lancet Oncol..

[13] Sakakibara R, Honda T, Mitsumura T, Kirimura S, Okubo K, Miyazaki Y (2021). Exploratory factors relevant to the success of the Oncomine™ Dx Target Test^Ⓡ^. Jpn. J. Lung Cancer.

[14] Taton O, Bondue B, Gevenois PA, Remmelink M, Leduc D (2018). Diagnostic yield of combined pulmonary cryobiopsies and electromagnetic navigation in small pulmonary nodules. Pulm. Med..

[15] Imabayashi T, Uchino J, Yoshimura A, Chihara Y, Tamiya N, Kaneko Y, Yamada T, Takayama K (2019). Safety and usefulness of cryobiopsy and stamp cytology for the diagnosis of peripheral pulmonary lesions. Cancers.

[16] Yarmus LB, Semaan RW, Arias SA, Feller-Kopman D, Ortiz R, Bösmüller H, Illei PB, Frimpong BO, Oakjones-Burgess K, Lee HJ (2016). A randomized controlled trial of a novel sheath cryoprobe for bronchoscopic lung biopsy in a porcine model. Chest.

[17] Nasu S, Okamoto N, Suzuki H, Shiroyama T, Tanaka A, Samejima Y, Kanai T, Noda Y, Morita S, Morishita N (2019). Comparison of the utilities of cryobiopsy and forceps biopsy for peripheral lung cancer. Anticancer Res..

[18] Sryma PB, Mittal S, Madan NK, Tiwari P, Hadda V, Mohan A, Guleria R, Madan K (2021). Efficacy of radial endobronchial ultrasound (R-EBUS) guided transbronchial cryobiopsy for peripheral pulmonary lesions (PPL's): A systematic review and meta-analysis. Pulmonology.

[19] Schuhmann M, Bostanci K, Bugalho A, Warth A, Schnabel PA, Herth FJF, Eberhardt R (2014). Endobronchial ultrasound-guided cryobiopsies in peripheral pulmonary lesions: A feasibility study. Eur. Respir. J..

[20] Kozak EA, Brath LK (1994). Do ‘screening’ coagulation tests predict bleeding in patients undergoing fiberopticbronchoscopy with biopsy?. Chest.

[21] Bjørtuft Ø, Brosstad F, Boe J (1998). Bronchoscopy with transbronchial biopsies: Measurement of bleeding volume and evaluation of the predictive value of coagulation tests. Eur. Respir. J..

[22] Kho SS, Chan SK, Yong MC, Tie ST (2019). Performance of transbronchial cryobiopsy in eccentrically and adjacently orientated radial endobronchial ultrasound lesions. ERJ Open Res..

[23] Jennings LJ, Arcila ME, Corless C, Kamel-Reid S, Lubin IM, Pfeifer J, Temple-Smolkin RL, Voelkerding KV, Nikiforova MN (2017). Guidelines for validation of next-generation sequencing-based oncology panels: A joint consensus recommendation of the Association for Molecular Pathology and College of American Pathologists. J. Mol. Diagn..

[24] Chen H, Luthra R, Goswami RS, Singh RR, Roy-Chowdhuri S (2015). Analysis of pre-analytic factors affecting the success of clinical next-generation sequencing of solid organ malignancies. Cancers (Basel).

[25] Nishii Y, Yasuma T, Ito K, Suzuki Y, Watanabe F, Kobayashi T, Nishihama K, D'Alessandro-Gabazza CN, Fujimoto H, Gabazza EC (2019). Factors leading to failure to diagnose pulmonary malignant tumors using endobronchial ultrasound with guide sheath within the target lesion. Respir. Res..

[26] Oki M, Saka H, Kogure Y, Niwa H, Yamada A, Torii A, Kitagawa C (2022). Ultrathin bronchoscopic cryobiopsy of peripheral pulmonary lesions. Respirology.

[27] Tanaka M, Matsumoto Y, Imabayashi T, Kawahara T, Tsuchida T (2022). Diagnostic value of a new cryoprobe for peripheral pulmonary lesions: A prospective study. BMC Pulm. Med..

[28] Oberg CL, Lau RP, Folch EE, He T, Ronaghi R, Susanto I, Channick C, Tome RG, Oh S (2022). Novel robotic-assisted cryobiopsy for peripheral pulmonary lesions. Lung.

[29] Katsis J, Roller L, Aboudara M, Pannu J, Chen H, Johnson J, Lentz RJ, Rickman O, Maldonado F (2021). Diagnostic yield of digital tomosynthesis-assisted navigational bronchoscopy for indeterminate lung nodules. J. Bronchol. Interv. Pulmonol..

